# The Impact of COVID-19 on Academic Cancer Clinical Trials in Canada and the Initial Response from Cancer Centers

**DOI:** 10.3390/curroncol29040197

**Published:** 2022-03-30

**Authors:** Stephen Sundquist, Diana Kato, Rebecca Y. Xu, James Schoales, Saranya Kulendran, Janet E. Dancey

**Affiliations:** 1 Canadian Cancer Clinical Trials Network, Toronto, ON M5G 0A3, Canada; diana.kato@oicr.on.ca (D.K.); rebecca.xu@oicr.on.ca (R.Y.X.); james.schoales@oicr.on.ca (J.S.); saranya.kulendran@oicr.on.ca (S.K.); janet.dancey@oicr.on.ca (J.E.D.); 2 Canadian Cancer Trials Group, Queen’s University, Kingston, ON K7L 3N6, Canada

**Keywords:** Canadian cancer clinical trials, academic cancer trials, COVID-19 pandemic impact, trial site activations

## Abstract

The COVID-19 pandemic resulted in temporary holds placed on new trial startups, patient recruitment and follow up visits for trials which contributed to major disruptions in cancer center trial unit operations. To assess the impact, the Canadian Cancer Clinical Trials Network (3CTN) members participated in regional meetings and a survey to understand the impact of the pandemic to academic cancer clinical trials (ACCT) activity, cancer trial unit operations and supports needed for post-pandemic recovery. Trial performance and recruitment data collected from 1 April 2020–31 March 2021 was compared to the same period in previous years. From 1 April–30 June 2020, patient recruitment decreased by 67.5% and trial site activations decreased by 81% compared to the same period in 2019. Recovery to reopening and recruitment of ACCTs began after three months, which was faster than initially projected. However, ongoing COVID-19 impacts on trial unit staffing and operations continue to contribute to delayed trial activations, lower patient recruitment and may further strain centers’ capacity for participation in academic-sponsored trials.

## 1. Introduction

The Canadian Cancer Clinical Trials Network (3CTN, the “Network”) was established in 2014 to improve recruitment, access and conduct of Canadian academic-sponsored, multi-center cancer clinical trials (ACCTs) [1]. 3CTN is currently comprised of 57 adult and pediatric cancer centers and supports an extensive ACCT Portfolio. In each year since 2014, Network cancer centers realized successive increases in overall recruitment to 3CTN Portfolio trials, reaching 148% above pre-3CTN baseline (i.e., 2.5× baseline) until March 2020 when the COVID-19 pandemic led to lockdown restrictions in all Canadian provinces resulting in interruptions in clinical trial conduct [2].

Across Canada, temporary holds were placed on new trial initiations and new patient accruals. Follow up visits for patients on trials were limited due to restricted access to health care facilities. Clinical research staff were redeployed to support patient care and/or COVID-19 research and had limited on-site access which contributed to major disruptions in cancer center trial unit operations. Public health and safety measures that continued through successive peaks in COVID-19 cases led to changes in the way clinical trials were conducted nationally and internationally [3,4,5]. Throughout this period, the 3CTN community discussed and implemented adaptive strategies to mitigate common challenges faced by trial units and to support post-pandemic recovery.

This report summarizes trial activities and performance data from 3CTN adult cancer centers and illustrates the impacts to ACCT activity in Canada between 1 April 2020 and 31 March 2021.

## 2. Methods

Trial units had to review and implement directives from regional health authority, institution and trial sponsors in response to the pandemic. To facilitate planning and implementation, 3CTN centers members held regional meetings to discuss requirements and impacts on trial operations. The minutes of these meetings were subsequently reviewed for this analysis. In April 2020, a survey was distributed to the centers to gather details of existing and projected impacts on academic trial recruitment and recommendations for Network-level support that would benefit post-pandemic recovery of the academic cancer trial ecosystem. Portfolio trial performance and recruitment data were collected from 1 April 2020–31 March 2021 and compared to the same period in each of the preceding two years (1 April 2018–31 March 2019 and 1 April 2019–March 2020). Data definitions and fields for Network center reporting of COVID-19-related holds affecting trial site setup, recruitment and follow up were created to enable tracking of trial status changes over time. A snapshot showing the status of Portfolio trials and cancer center trial operations was taken on June 2020.

## 3. Results

Reports on the COVID-19 impact were obtained during regional meetings from 67% (28/42) of member centers in attendance and from 85% (36/42) of centers that responded to the survey. A total of 41/42 adult member centers submitted quarterly recruitment and trial performance data to 3CTN from 1 April 2020–31 March 2021.

### 3.1. Trial Recruitment and Activation of New Trials

Across Canada, centers reported that recruitment to interventional trials addressing essential research (e.g., lifesaving treatment in areas of unmet need) had continued, while recruitment to the majority of other trials were placed on hold for an indefinite period. Initial reports also consistently noted that new trial activations were halted, with exceptions for COVID-19-related research or novel therapy trials.

Survey results from April 2020 showed that 69% (25/36) of centers had more than half of their Portfolio trials on hold to patient recruitment. By June 2020, the percentage of Portfolio trials on hold had dropped to 51.8% (262/505), based on reporting from 83% (35/42) of centers. Furthermore, the outlook on the prospect for recovery at that time was relatively optimistic with 57% (21/37) of centers forecasting that >75% of Portfolio trials would reopen to patient recruitment by the end of the year.

**Figure 1:**
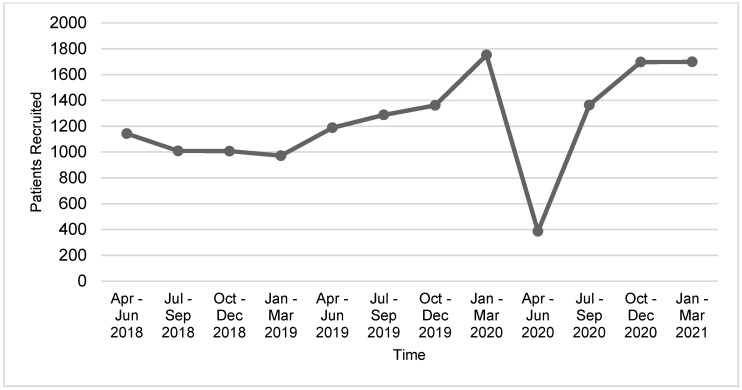
Recruitment to 3CTN Portfolio trials at adult cancer centers (*n* = 41) from April 2018–March 2021.

**Figure 2:**
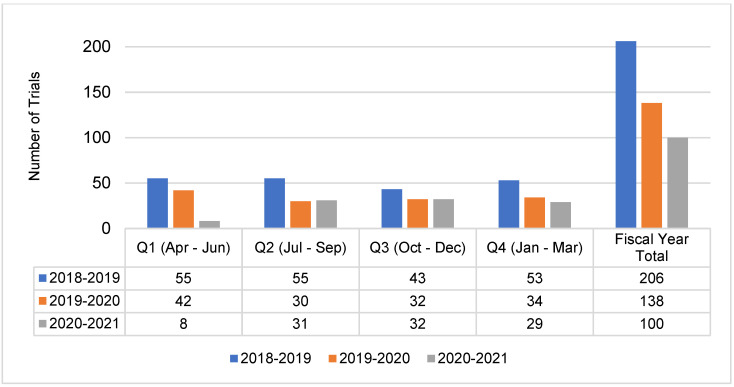
Trial site activations at adult Network centers (*n* = 41) from 1 April 2018–31 March 2021.

**Figure A1:**
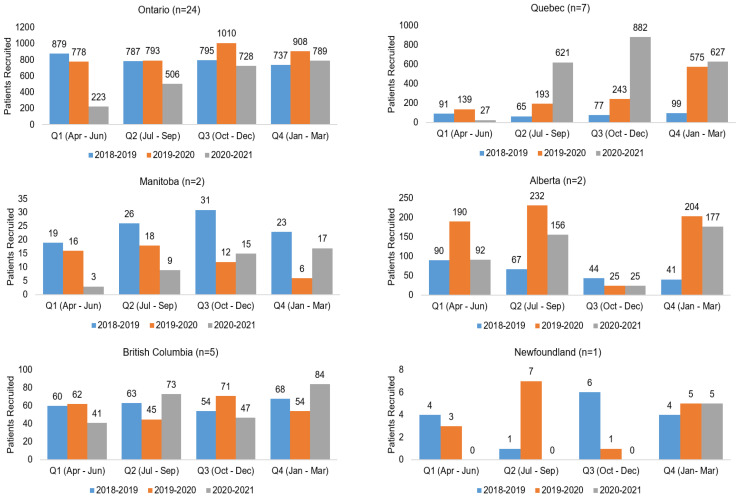
Recruitment to 3CTN Portfolio trials at adult cancer centers by province 1 April 2018–31 March 2021.

**Figure A2:**
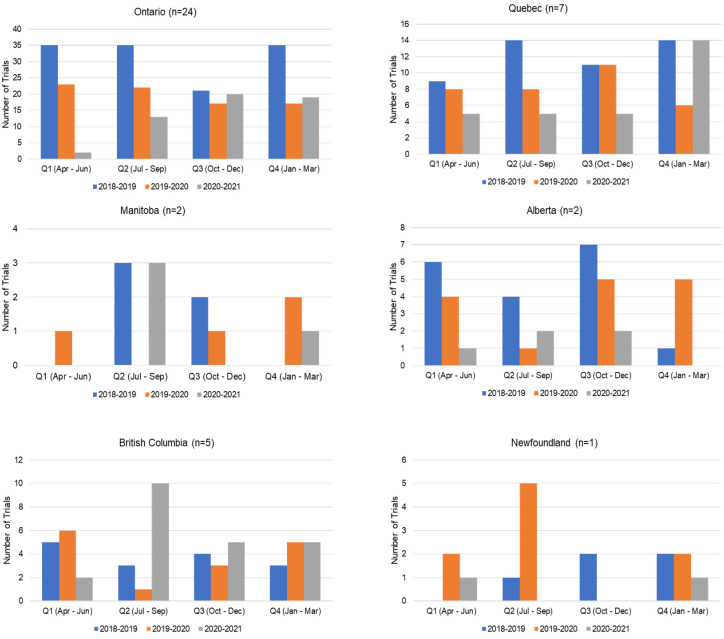
3CTN Portfolio trial site activations at adult cancer centers by province from 1 April 2018–31 March 2021.

Numbers of trial activations and patient recruitment data for the year spanning 1 April 2020–31 March 2021 were reviewed and compared to pre-pandemic performance data. Declines in trial activity were most pronounced during the first quarter period, 1 April–30 June 2020, where patient recruitment to Portfolio trials at the 41 reporting adult cancer centers decreased by 67.5% and trial activations decreased by 81% compared to the same period in 2019 (Figure 1 and Figure 2). Compared to the same 12-month period in the previous year, overall recruitment to Portfolio trials and trial activations at cancer centers decreased by 48% and 27.5%, respectively. The recovery period varied by province (Figure A1 and Figure A2), reflecting differences in onset of peaks in COVID-19 cases, implementation and easing of public health and institution restrictions on trial activities.

### 3.2. Delays to Post-COVID-19 Recovery

Reported reasons for delays to recovery (defined as removal of restrictions to trial activation and new patient recruitment) at clinical trial centers were several. These included institutional-level decisions and priorities for trials, access to auxiliary services (e.g., pharmacy, imaging, laboratory), high staff turnover and clinician fatigue affecting capacity for trial activities. Study sponsors required addressing emergent matters impacting patient management and protocol compliance before reopening of trials to new patient recruitment. Timelines for activation of new trials were impacted jointly by resource constraints at the sponsor and individual site level.

### 3.3. Network Support Provided to Centers

3CTN members provided information on key needs to address impacts and promote recovery of ACCT activities. Based on information provided by Network members, several measures were undertaken to mitigate some of the rapid changes to clinical trial conduct introduced by the pandemic. This included launching a new webinar series offering peer-to-peer support for centers implementing remote monitoring and patient consent procedures. Furthermore, changes to the Network’s funding model for the coming year were designed with input from members to promote rapid recovery of academic trials by providing incentives for new ACCT activation and accrual start, as well as for undertaking remote patient recruitment and management.

## 4. Discussion

Across 3CTN, measures were implemented to capture COVID-19 impacts on the ACCT trial activities and member centers. The information obtained from status reports during Network meetings, surveys and trial performance data collected throughout a 12-month period indicated that recovery to reopening and recruitment of active trials to pre-pandemic levels occurred following an initial three-month period (April–30 June 2020). Similarly, after a significant decline seen in the first quarter, new trial activations for the remainder of the year were comparable to that from the previous year. This recovery was faster than predicted by the majority of Network members.

Recovery was supported by 3CTN activities that mobilized the trial community to address COVID-19 impacts on ACCTs, and revised funding incentives created to promote activation and patient accrual to new Portfolio trials,. Activities included quarterly Network meetings and a webinar series that supported peer-to-peer knowledge exchange and collaborative problem-solving. Meetings resulted in shared awareness and interpretation of emerging developments, successful mitigation strategies and best practices for adapting to a rapidly changing regulatory and operating environment. Continued national and regional progress towards recovery and the emergence of longer-term impacts affecting the Canadian academic cancer clinical trial landscape will be monitored across the Network.

Despite these overall favorable results, the recovery of academic clinical trial activity was not uniform across the Network. This is likely attributable to regional variation in COVID-19 incident rates, institution and public perception of trial participation and local capacity for trial activity. Local public health and institutional response measures limited patient and non-essential staff access to centers, resulting in decreased patient accrual and requiring process changes so that trial activities could continue. As measures were lifted, some Network centers found patients unwilling to participate in trials due to safety concerns. As oncology clinical trial staff were redeployed to support COVID-19 activities at their institutions, the operational burden of clinical trial programs fell to remaining staff. Concurrently, site staff had to implement new processes to assess patients, to address regulatory compliance and study-specific directives from sponsors. Increased rates of staff burnout and turnover have also been widely reported by centers.

There are a number of limitations to this study. Data limitations include incomplete reporting and survey responses received from centers. Reported impacts to trial unit operations and trial activity were provided during quarterly Network status meetings and captured in meeting summaries rather than structured interviews. In addition, data may not be comparable across regions due to variations peak COVID-19 incidences and recovery times, and the differences in capacities to manage trial patients and activities at any given point during the reporting period.

Despite these limitations, the data presented provide a summary of academic trial experience through COVID-19 and suggest that there was a profound initial impact followed by early signs of recovery throughout much of Canada. Widespread and effective vaccination of Canadians has supported the recovery of clinical trial activities toward pre-pandemic levels [6]. However, there are concerns that an already limited capacity for some centers to participate in academic sponsored trials due to rising costs may be further strained by ongoing and post-COVID-19 impacts. Over the short term, trial units must address the impact of reduced trial activities on trial unit revenues, particularly for those centers that have seen slower recovery. Activation and recruitment to higher-paying industry sponsored trials may be prioritized over new academic trial opportunities.

As health system capacity increases and addresses COVID-19-related delays in diagnosis and treatment, there will be increases in new cancer diagnoses and more advanced cancers requiring urgent treatment. Access to clinical trials will remain a priority; however, trial eligibility, availability and staff capacity must be sufficient to ensure recruitment [7]. Trial staff will also be stretched to address the backlog of trial monitoring and auditing activities delayed due to COVID-19 restrictions. Accrual and new trial activities will be assessed by 3CTN in the coming years to identify changes in the metrics and determine additional infrastructure and funding support for Canadian cancer centers necessary to support ACCT activities. Comparisons of disease incidence rates to data summarizing newly opened trials by disease type and other attributes may also highlight areas where investments in new research may be required.

In summary, we found that while 3CTN member centers were successful in adjusting to immediate COVID-19 impacts on ACCTs, the recovery of the Canadian trial environment is incomplete and is variable. Planned review of reported trial activity and performance data for current and coming years will provide added insight into the recovery progress across member cancer centers, including the impact of the most recent Omicron variant outbreak. Further restoration of Canada’s academic trial capacity will benefit from an ongoing, coordinated Network response.

## Data Availability

The data presented in this study are contained within this article.
